# *Cutibacterium acnes* Biofilm Study during Bone Cells Interaction

**DOI:** 10.3390/microorganisms8091409

**Published:** 2020-09-12

**Authors:** Céline Mongaret, Jennifer Varin-Simon, Fabien Lamret, Taghrid S. El-Mahdy, Lucien Brasme, Véronique Vernet-Garnier, Sophie C. Gangloff, Xavier Ohl, Fany Reffuveille

**Affiliations:** 1 EA 4691 Biomatériaux et Inflammation en Site Osseux (BIOS), Université de Reims Champagne-Ardenne, SFR Cap Santé (FED 4231), 51100 Reims, France; jennifer.varin-simon@univ-reims.fr (J.V.-S.); fabien.lamret@univ-reims.fr (F.L.); sata186@hotmail.com (T.S.E.-M.); sophie.gangloff@univ-reims.fr (S.C.G.); xohl@chu-reims.fr (X.O.); fany.reffuveille@univ-reims.fr (F.R.); 2 Service Pharmacie, Centre Hospitalier Universitaire de Reims (CHU Reims), 51100 Reims, France; 3 Department of Microbiology and Immunology, Faculty of Pharmacy, Helwan University, Cairo 11795, Egypt; 4 Department of Microbiology, Faculty of Pharmacy, Ahram Canadian University, Cairo 12585, Egypt; 5 Laboratoire de Bactériologie-Hygiène, Centre Hospitalier Universitaire de Reims (CHU Reims), 51100 Reims, France; lbrasme@chu-reims.fr (L.B.); vvernetgarnier@chu-reims.fr (V.V.-G.); 6 Service d’Orthopédie et Traumatologie, Centre Hospitalier Universitaire de Reims (CHU Reims), 51100 Reims, France

**Keywords:** joint infections, biofilms, host–pathogen interactions, *Cutibacterium acnes*, internalization

## Abstract

*Cutibacterium acnes* is an opportunistic pathogen involved in Bone and Prosthesis Infections (BPIs). In this study, we observed the behavior of commensal and BPI *C. acnes* strains in the bone environment through bacterial internalization by osteoblast-like cells and biofilm formation. For the commensal strains, less than 1% of the bacteria were internalized; among them, about 32.7 ± 3.9% persisted intracellularly for up to 48 h. *C. acnes* infection seems to have no cytotoxic effect on bone cells as detected by LDH assay. Interestingly, commensal *C. acnes* showed a significant increase in biofilm formation after osteoblast-like internalization for 50% of the strains (2.8-fold increase). This phenomenon is exacerbated on a titanium support, a material used for medical devices. For the BPI clinical strains, we did not notice any increase in biofilm formation after internalization despite a similar internalization rate by the osteoblast-like cells. Furthermore, fluorescent staining revealed more live bacteria within the biofilm after osteoblast-like cell interaction, for all strains (BPIs and commensal). The genomic study did not reveal any link between their clinical origin and phylotype. In conclusion, we have shown for the first time the possible influence of internalization by osteoblast-like cells on commensal *C. acnes.*

## 1. Introduction

*Cutibacterium acnes* is a commensal bacterium playing an important role in the ecosystem of healthy human skin. However, this species is also recognized as a pathogen in foreign body infection: endocarditis, prostatitis and specifically in Bone and Prosthesis Infections (BPIs) [1], for which *C. acnes* has an incidence of approximately 10% (more frequently in the shoulder, the hip, the spine and the knee joints) [2,3]. *C. acnes* strains responsible for BPIs mostly originate from the patient’s own normal skin microbiota [4]. *C. acnes* is still isolated after surgery despite skin disinfection [5]. The hypothesis is that *C. acnes* hide in the hair bulbs and are spilled during surgical incision.

*C. acnes* infections do not present obvious clinical symptoms, which appear more than 3 months after surgery [1,2]. Low-grade clinical symptoms and specific bacteriological culture conditions, a prolonged incubation time in anaerobic conditions of growth, demonstrate that its clinical diagnosis is challenging. Indeed, *C. acnes* infections do not induce a clear inflammatory response, meaning this microorganism is able to escape the immune system, probably thanks to the bacterial internalization by host cells [6]. Aubin et al. revealed that *C. acnes* isolates from BPIs were internalized by human osteoblast-like cells [7]. These hidden bacteria in osteoblast-like cells could explain the chronicity of BPIs, an event already described by Josse et al. with *Staphylococcus aureus*, species mainly involved in BPIs. The internalization by osteoblasts is a key element to protect *S. aureus* from the immune system and to sustain the infection [8]. From this observation, the internalization by osteoblast-like cells could support the hypothesis of an increased resistance of *C. acnes* to immune system attacks or antibiotics [8].

*C. acnes* infections are difficult to treat, as this anaerobic bacillus is able to form biofilms on surgical material because the host rapidly coats the surface of an implant with extracellular matrix proteins on which bacteria can easily adhere, which is the first step of biofilm formation [9]. The main problem of *C. acnes* biofilm is that the embedded bacteria are less susceptible to antimicrobials than their planktonic counterparts as the biofilm is a complex bacterial community with altered phenotypes surrounded by a protective matrix composed of extracellular DNA, protein and polysaccharides [10]. Moreover, the *C. acnes* biofilm formation also increases the putative virulence factor expression [6,11]. Holmberg et al. observed that isolates from deep infections produced more biofilm than skin isolates, highlighting the role of biofilms in *C. acnes* virulence, regardless of clinical origin [12].

Indeed, *C. acnes* could be classified by its clinical origins, its virulence phenotype and also by its genomic evolution. *C. acnes* is subdivided into six main phylotypes: IA1, IA2, IB, IC, II and III. Mac Dowel et al. has correlated several phylotypes of *C. acnes* with several clinical pathways [13]. Indeed the various subgroups play a significant role in different skin disorders [14]. For example, Paugam et al. demonstrated that phylotype IA1 is predominant in acne lesions in clinical dermatology, a result found after performing a prospective observational study [15]. More recently, Kilian et al. have highlighted the presence of subgroups in the phylotypes called multilocus sequence typing (MLST), according to a genome analysis [16]. Using different lineages of *C. acnes*, Aubin et al. investigated the MLST profile and *C. acnes* host–pathogen interaction in BPIs, identifying two *C. acnes* behaviors depending on their genetic background clone complex (CC), although these groups are not correlated to clinical origin [7]. A correlation between genomic (MLST profile) and proteomic (SELDI-TOF profile) data was previously described by Dekio et al., suggesting that the microenvironment of each *C. acnes* subtype may influence protein expression [17]. All these previous studies suggest that *C. acnes* virulence behavior is linked to the clinical origin or environment without any correlation with genetic background.

In this study, we hypothesized that bone environment, especially internalization by host cells, could induce *C. acnes* pathogenesis. We analyzed the behavior of clinical commensal *C. acnes* isolates and then compared them to the BPI isolates before and after internalization by osteoblast-like cells. Finally, we investigated their phylotype and CC profile to identify a potential link between genetic background and virulence behaviors.

## 2. Materials and Methods

### 2.1. Culture Media

The human osteosarcoma cell line SaOS2 purchased from ATCC (ATCC^®^ HTB 85^TM^) was cultured in a 5% CO2 atmosphere at 37 °C in Dulbeccco’s Modified Eagle Medium (DMEM, Gibco, Invitrogen, Carlsbad, CA, USA) supplemented with 10% of fetal bovine serum (Gibco, Invitrogen, Carlsbad, CA, USA) and a 1% antibiotic PenStrep solution (Gibco, Invitrogen, Carlsbad, CA, USA). The medium was changed every two days.

### 2.2. Bacteria Strains Culture

*Cutibacterium acnes* (formerly *Propionibacterium acnes*) strains were isolated and anonymized at the laboratory of bacteriology of Reims University hospital (CHU Reims). Fifteen strains were isolated by swabbing from fifteen different patients: nine BPIs and six commensal isolates. *C. acnes* BPIs were defined when at least three of the five samples from the bone and joint tissue during orthopedic surgery were positive. The positivity was discussed and confirmed by the consultation of an expert multidisciplinary team (named CRIOA: national and referent center for bone and joint infections). When less than three *C. acnes* positive samples were identified from the samples, which were not from bone and joint tissue, the strains were defined as commensal strains. Implant-associated infections were confirmed using the Infectious Diseases Society of America criteria for bone and joint infections [16]. The clinical source of the isolates is mentioned in Table 1. The reference strain ATCC 6919 was isolated from facial acne and used as the referent strain to identify the variability of the *C. acnes* virulence strains with a different clinical origin. *C. acnes* strains were isolated on Columbia agar with 5% sheep blood (BioRad, Hercules, CA, USA) and cultivated in a Brain Heart Infusion (BHI) broth (BioRad, Hercules, CA, USA) for five days under anaerobic conditions using the GenBox system (Biomerieux, Marcy l’Etoile, France) at 37 °C.

### 2.3. C. acnes Internalization by Osteoblast-Like Cells

Bacterial internalization experiments were adapted from Josse et al.’s study [18]. Briefly, 10.5 × 10^3^ SaOS2 cells/cm^2^ were seeded in 24-well culture plates and were incubated at 37 °C for 72 h. Cell cultures were then washed with a phosphate-buffered saline solution (PBS, Gibco, CA, USA) and incubated overnight with 1 mL of media without antibiotics. The following day, cells were washed with PBS and incubated with 1 mL of media without antibiotics. One well was used for counting the number of cells per well. Bacteria were centrifuged for 5 min. at 5000× *g*, and the pellets were rinsed with PBS. Bacteria were then added to the culture medium to obtain a multiplicity of infection (MOI) of 100:1 (*C. acnes*: cell). After 3 h of interaction, the cells were washed with PBS and incubated with cell medium containing 100 µg/mL of gentamicin (Fisher Scientific, Hampton, NH, USA), during 1 h at 37 °C, in a 5% CO2 humidified atmosphere. Then, cells were washed again with PBS and a 0.1% Triton X-100 (Sigma, Saint Louis, MO, USA) solution was added to each well to harvest the intracellular bacteria. Lysates were seeded on blood agar plates using easySpiral (Intersciences, St Nom de la Breteche, France) at 37 °C under anaerobic condition, using the GenBag system for 5 days to determinate the number of recovered colony-forming units (CFU). The percentage of bacteria was calculated as follows: % of bacteria = (CFU/mL × 100)/(number of cells × MOI). Each experiment was done three (independent) times and with at least two technical repeats (at least six raw data points were used in the statistical analysis).

### 2.4. LDH Measurement Assay

To determine the bacterial impact on the osteoblast-like cells, the lactate dehydrogenase (LDH) activity was evaluated with a cytotoxicity detection kit (Roche, Bâle, Switzerland), following the manufacturer’s instruction. Briefly, after 3 h of cells/bacteria interaction and after 48 h post-infection, supernatants were collected and filtered to remove cells debris and bacteria. Then, they were incubated with a substrate during 15 min in the dark and absorbance was measured at 490 nm with a correction at 700 nm. Each experiment was done three (independent) times and with at least two technical repeats (at least six raw data points).

### 2.5. Static Biofilm Model (Crystal Violet Staining Model)

As previously described, the biofilm biomass was evaluated by crystal violet staining [19,20]. An isolated colony of *C. acnes* was diluted in 1 mL of BHI medium in a 48-well plastic microtiter plate or in presence of a titanium piece. After 5 days of anaerobic incubation at 37 °C, the planktonic growth was evaluated by measuring the absorbance at 600 nm. The plates were gently washed 3 times with water and 1 mL of 0.18% crystal violet was applied for 20 min. After washing with water, 1 mL of 95% ethanol was added to each well and thus the stained biofilm was evaluated by measuring the absorbance at 595 nm. All results are expressed with the subtraction of the blank: medium without bacteria. Each experiment was done three (independent) times and with at least three technical repeats (at least nine raw data points).

### 2.6. Fluorescent Microscopy

Biofilms were stained using the LIVE/DEAD BacLight Bacterial Viability kit (Molecular Probes, Eugene, OR) for the microscopy experiments. A ratio of SYTO-9 (green fluorescence, all live and dead cells) to propidium iodide (PI) (red fluorescence, damaged bacterial membrane cells or “dead” bacteria) of 1:5 was used. Image acquisitions were performed on an Axiovert 200M inverted microscope using a ×40 objective and the dedicated Axiovision v 3.2.6 software (Carl Zeiss, Oberkochen, Germany). Equal acquisition times were set for each SYTO-9 and PI channel in any condition of all the experiments. Surface quantification of live and dead bacteria was determined using ImageJ software (v1.50i, National Institutes of Health, Bethesda, MD, USA). Each experiment was done three (independent) times and with at least two technical repeats (at least six raw data points).

### 2.7. Persistence of Biofilm

After internalization, bacteria were seeded at different passages on blood agar plates or on a BHI solution to observe the persistence of the biofilm. From Culture 1 (P1 = bacteria seeded after 3 h and Gentamicin treatment after internalization) to Culture 5, PO represents the bacteria culture before internalization. Biofilm formation was measured by crystal violet staining as described above. Each experiment was done three (independent) times and with at least three technical repeats (at least nine raw data points).

### 2.8. Phylotype and MLST Data Analysis

Phylotype and MLST were performed according to the protocol available at https://pubmlst.org/pacnes/ and the clonality was carried out by MLST on all isolates as previously described [20]. Briefly, bacterial DNA was extracted using the QIAmp DNA Mini kit (Qiagen, Hilden, Germany) according to the manufacturer’s instruction. Bacterial genes *aroE, atpD, guaA, lepA, sodA, tly* and *camp2* were amplified by end-point PCR. DNA amplification samples and sequencing primers were sent to the Genewiz society for sequencing bacteria. For each gene, the DNA sequences were entered into the “*Cutibacterium acnes* MLST database” to identify the corresponding allele. All gene alleles were recorded in the database and, for each *C. acnes* strain, a phylotype and MLST profile were automatically attributed by the pubmlst database.

### 2.9. Statistical Methods

All values are the means of at least three independent experiments (three biological repeats). Moreover, each experiment was done at least in two (internalization experiment) or three (crystal violet model) technical repeats (at least six to nine raw data points). The statistical significance of the results was assessed using non-parametric analysis with pairwise tests. The exact non-parametric Wilcoxon–Mann–Whitney test for independent samples was used (StatXact 7.0, Cytel Inc). Non-parametric statistics were used owing to the lack of a normal distribution of the assessed variables. Stratification allowed the impact of technical variability to be taken into account. Differences were considered significant at *p* < 0.05.

## 3. Results

### 3.1. Commensal C. acnes Strain Behaviors

*C. acnes* strains isolated from patients who did not declare *C. acnes* infection (C “commensal” strains) were studied for their capacity to form biofilms: five strains belonged to the IAI phylotype and one to the IC phylotype, according to the MLST assay (Table 1).

**Figure 1:**
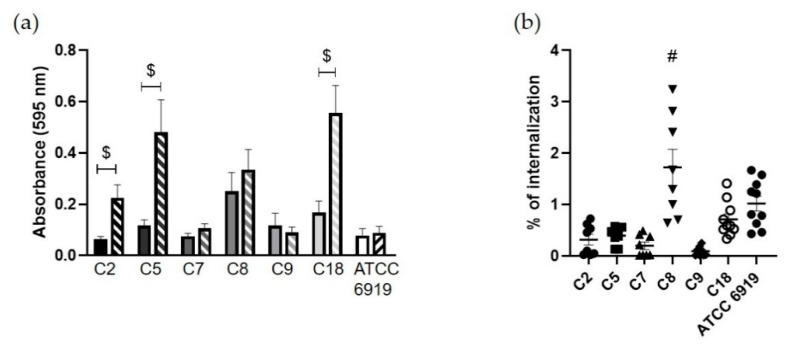
Behaviors of the commensal *C. acnes* strains. (**a**) Biofilm forming capacity by crystal violet quantification before (full bars) and after internalization (hatched bars) by osteoblast-like cells ($; *p* < 0.05 compared to initial biofilm quantification); (**b**) internalization rate (%) by osteoblast-like cells (#; *p* < 0.05; different to all other strains).

The crystal violet staining assay allowed quantifying the *C. acnes* biofilm biomass. After 5 days of biofilm development, we observed that the six commensal strains formed low amounts of biofilm with an average absorbance of 0.13 ± 0.02 for the C strains (from a value of 0.06 for C2 to a value of 0.25 for C8; Figure 1a), also noticed for the lab strain ATCC 6919.

We have not noticed a significant difference in planktonic growth for all the tested strains before and after internalization by osteoblast-like cells (Figure S1).

Then, we evaluated the rate of internalization after 3 h of co-culture of these strains by osteoblast-like cells (SaOS2). The mean percentage of internalized commensal bacteria by osteoblast-like cells was 0.58 ± 0.1% (Figure 1b). Strain C8 had an internalization rate of 1.72 ± 0.35%, which was significantly higher and more variable than the other strains. As observed through LDH assay quantification, the interaction between *C. acnes* and osteoblast-like cells for three hours had no consequence on cell cytotoxicity (Figure S2).

To determine if the internalization could have a specific impact on *C. acnes* capacity to form a biofilm, internalized bacteria were collected and cultured to monitor the biofilm formation after 5 days of incubation. We observed an average absorbance of 0.368 ± 0.04 for all commensal strains, from 0.09 for C9 to 0.56 for strain C18, which possessed the highest biofilm coloration. Furthermore, comparing the quantity of formed biofilm by *C. acnes* before (Figure 1a) and after the internalization (Figure 1a), a significant increase in biofilm formation was revealed after internalization for three *C. acnes* commensal strains, C2, C5 and C18, (with fold-increase rates of 3.83, 4 and 3.3; respectively) and a non-significant increase for one strain (C8 with a fold-increase of 1.4).

**Figure 2:**
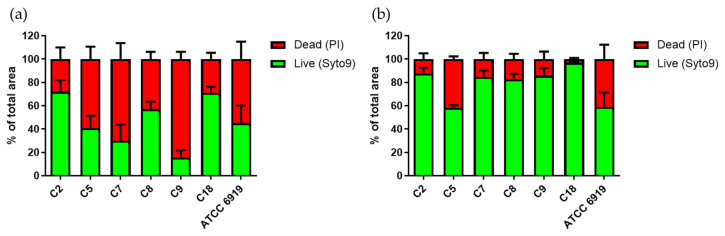
The living bacterial proportion in the commensal *C. acnes* strain biofilms. Repartition of Syto9 (green bars) and PI (red bars) staining within biofilms formed by the commensal *C. acnes* strains before (**a**) and after internalization (**b**) by osteoblast-like cells. Acquisition of images by fluorescent microscopy and calculation by Image J software.

To deepen this approach, using live/dead fluorochromes, the percentage of Syto9 and PI (damaged bacterial membrane or “dead” bacteria) staining was determined in the fluorescent images of the initial and post-internalization biofilm of the commensal strains (Figure 2). We observed a mean proportion of 50.4% of live bacteria before internalization (values from 16 to 72% according to the strains), which increased to 80.6% after internalization (*p* < 0.05; values from 58 to 97%).

### 3.2. Commensal C. acnes Fate after Internalization

**Figure 3:**
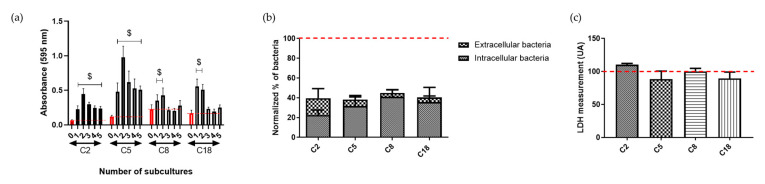
Fate of the internalized commensal *C. acnes*. (**a**) Stability of biofilm increase for intracellular *C. acnes* strains. Each time, red histograms represent the quantification of the initial biofilm formed before internalization. Other histograms have a number corresponding to the number of cultures after internalization ($, *p* < 0.05); (**b**) percentage of viable intracellular and extracellular bacteria following 48 h post-infection; 100% corresponding to intracellular *C. acnes* strains at 3 h of interaction (red dot line); (**c**) LDH release measurement normalized on cells without bacteria, in supernatant after 48 h of *C. acnes*/osteoblast-like cells interaction; 100% corresponding to the measurement of cells without bacteria.

The stability of the behavioral change after internalization for the three commensal *C. acnes* strains with significant modification (C2, C5 and C18) and the C8 strain, which possessed a tendency for a similar behavior, was tested by performing subcultures of these strains and by quantifying the formed biofilm after each culture (Figure 3a). We observed a stable increased biofilm amount for 2 of the 4 strains (C2 and C5), underlining a change in bacterial metabolism. After a 3h interaction and antibiotic treatment, we enumerated the quantity of the intracellular and extracellular bacteria after 48 h of co-culture to investigate the possibility of persistence of the *C. acnes* strains in osteoblast-like cells (Figure 3b).

We observed that a low percentage of initial number of bacteria quantified as intracellular after 3 h of interaction were externalized (3.9 ± 3% (C8) to 17.2 ± 9.6% (C2)). The majority of the bacteria were either still in the osteoblast-like cells (32.7 ± 3.9%) or supposedly died (> 50%) (Figure 3b). Furthermore, we tested the cytotoxicity effect of the internalized bacteria on the osteoblast-like cells. No difference in LDH measurement was observed between infected and non-infected cells (Figure 3c).

### 3.3. Behaviors Comparison with BPI C. acnes Strains

**Figure 4:**
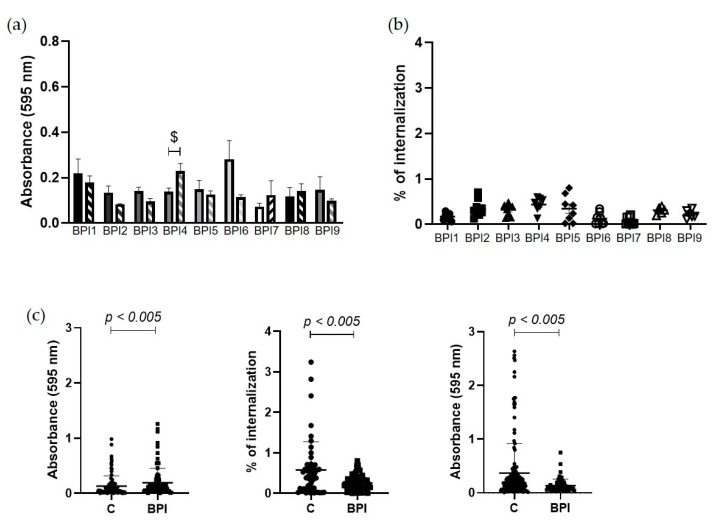
Behaviors of the *C. acnes* BPI strains and comparison with the commensal strains. (**a**) Biofilm forming capacity by crystal violet quantification without interaction (full bars) and after internalization (hatched bars) by osteoblast-like cells by crystal violet quantification $; *p* < 0.05 compared to initial biofilm quantification; (**b**) internalization rate (%) by osteoblast-like cells; (**c**) comparison commensal and BPI strains compiled results: behaviors of *C. acnes* commensal “C” strains and strains isolated from BPIs: biofilm quantification by crystal violet assay (left graph); internalization rate (%) by osteoblast-like cells (middle graph); and biofilm quantification after internalization (right graph).

*C. acnes* strains isolated from BPIs (shoulder prosthesis or osteosynthesis material infection) were studied for their capacity to initiate biofilms, to be internalized by osteoblast-like cells and to form biofilm post-internalization (Figure 4a,b). We have not noticed a significant difference in planktonic growth for all the tested strains before and after internalization by osteoblast-like cells (Figure S1), with the exception of BPI8.

Figure 4c illustrates the comparison between the commensal and BPI *C. acnes* strains. As for the commensal strains, the BPI strain genotype was determined by the MLST approach. Six BPI strains were identified with an IA1 phylotype: three were isolated from shoulders, two from the spine and one from the hip (Table 1). Three strains presented an IB phylotype: two of them were isolated from shoulder prostheses and one from tibia material.

After 5 days of biofilm development, we observed an absorbance average of 0.192 ± 0.03 with a lower value of 0.07 for BPI 7 and the highest value of 0.28 for BPI 6 (Figure 4a). All the BPI strains possessed a similar internalization rate with an average of 0.26 ± 0.02% (Figure 4b). Concerning the formed biofilm by intracellular bacteria (Figure 4a), no significant change was observed at the exception of strain BPI 4, which showed an increase in biofilm formation post-internalization (1.64-fold; *p* < 0.05). Nevertheless, in order to facilitate the comparison between the strains from a different origin of isolation (commensal “C” vs. BPI stains), the presented data were compiled (Figure 4c). Thus, the difference in behavior between the strains was highlighted in particular for the initial biofilm formation, which was slightly weaker for the commensal strains (0.13 ± 0.02) compared to the BPI strains (0.192 ± 0.03; *p* < 0.05). In addition, we noticed a significant difference with a higher internalization rate for the commensal strains compared to the BPIs (0.58 ± 0.1 vs. 0.26 ± 0.02, respectively; *p* < 0.05), while the data distribution was also wider for the commensal strains. Then, the commensal bacteria after internalization formed more biofilm than the BPI strains (0.37 ± 0.04 vs. 0.31 ± 0.01, respectively; *p* < 0.05) with very high values (absorbance of 3.0).

**Figure 5:**
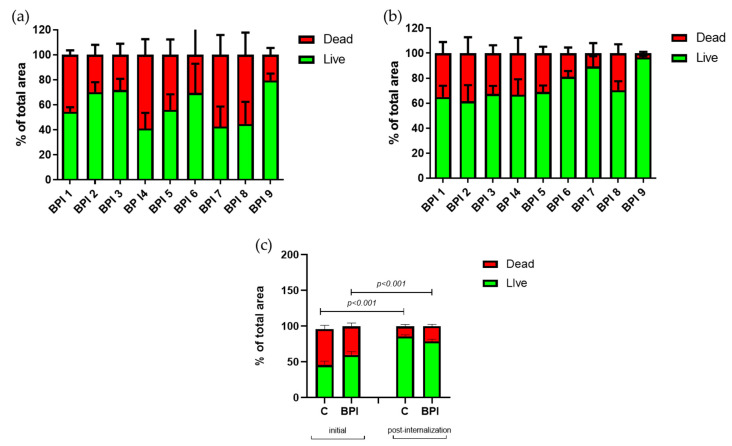
The living bacterial proportion in the BPI *C. acnes* strain biofilms and comparison with the commensal strain biofilms. Repartition of Syto9 (green bars) and PI (red bars) staining within biofilm formed by the *C. acnes* strains isolated from BPIs before (**a**) and after internalization (**b**) by osteoblast-like cells; (**c**) comparison of commensal and BPI strains: live dead proportion quantification within biofilm of *C. acnes* commensal “C” strains and isolated from BPIs. Images acquisition by fluorescent microscopy and calculation by Image J software.

Concerning the live/dead proportion of bacteria within the biofilm (Figure 5a,b), each BPI strain had an increased proportion of live bacteria after internalization, except for BPI2. Figure 5c illustrated a mean proportion of 61.2% of BPI live bacteria before internalization (values from 41 to 82% according to the strains), which increased to 76.2% after internalization (values from 62 to 97%), *p* < 0.05. However, even if the proportion of live/dead bacteria is similar for all strains before and after internalization (Figure 5c), an increase is observed in the proportion of live bacteria of 25% for BPIs and 60% for commensal strains within the formed biofilm post-internalization (*p* < 0.05).

### 3.4. Towards In Vivo (Commensal Biofilm on Titanium)

**Figure 6:**
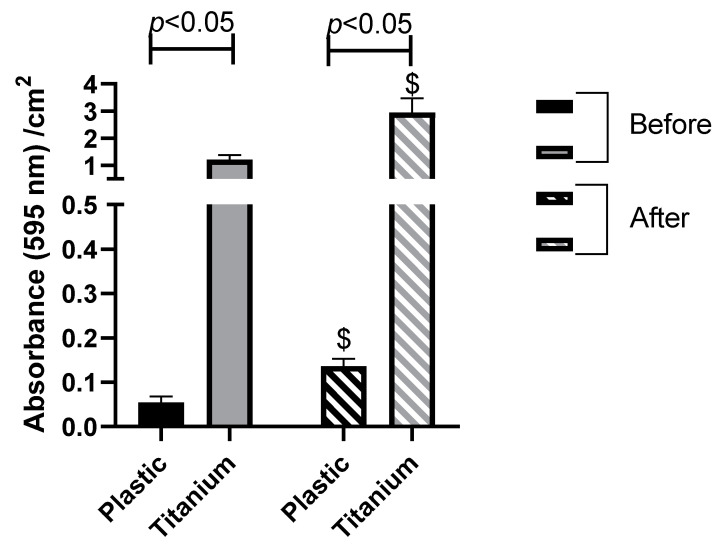
Comparison of the commensal strains’ adhesion (compiled results of the four strains) between plastic and titanium surfaces before and after internalization by osteoblast-like cells. Biofilm-forming capacity by crystal violet quantification ($, significant difference with formed biofilm before internalization, *p* < 0.01).

The biofilm formation of the four-selected commensal *C. acnes* strains on titanium was studied, as the main material used for the manufacturing of prostheses. We compared the adhesion of the commensal strains on plastic and on titanium (Figure 6). The biofilm-forming ability of our commensal strains is much higher on titanium surfaces than plastic regardless of interaction with SaOS2 cells. A 22- and 21.6-fold increase were observed for the formed biofilm on titanium vs. plastic, respectively, before and after internalization (Figure 6).

## 4. Discussion

In recent years, *Cutibacterium acnes* has been increasingly reported as one of the most frequently isolated pathogens in deep tissue infection, complicating shoulder surgery [2,21]. This commensal microbiota can somehow induce BPIs distant-time to the implantation surgery. The objective of this work was to evaluate the virulence of naive *C. acnes* strains (from commensal flora) in contact with bone cells to understand the physiopathology of these infections. For the first time, to our knowledge, we showed that the commensal bacterial internalization by osteoblast-like cells had an impact on their biofilm formation capacity and this effect was even amplified on a titanium support.

We showed that *C. acnes* was internalized by osteoblast-like cells (SaoS2), regardless of the clinical origin (commensal or BPIs). Some studies have already demonstrated how *C. acnes* can be internalized into cell lines, such as the prostate epithelial cell line (RWPE-1) [22] or human acute monocytic leukemia cell line THP-1 [23]. Our results are in accordance with Aubin et al., who revealed that *C. acnes* is also internalized by another bone cell line, MG63, and this phenomenon is not correlated to the clinical origin of the strains [24].

In this study, although there is a significant difference in the rate of internalization between commensal and BPIs, the overall rate of internalization by osteoblast-like cells is less than 1%, regardless of the clinical origin, in below the 30% internalization rate found in *S. aureus* [8].

However, we wondered if the commensal strains could persist or exit from the osteoblast-like cells after internalization. After interaction during 3 h and elimination of the extracellular bacteria, the internalized bacteria’s fate was analyzed in extending the culture of the infected cells for 48 h. We did not notice any cytotoxic effect on the infected osteoblast-like cells, contrary to *S. aureus* infections [19]. A minority part of *C. acnes* became extracellular (8.5 ± 2.9%), probably due to the mechanisms of the bone cells. More than 50% of the internalized bacteria were not found after 48 h, strongly assuming a high death rate (61.5%). One tierce of bacteria remained intracellular (32.7 ± 3.9%). This phenomenon of intracellular persistence may allow *C. acnes* to survive in a quiescent form that can be reactivated and proliferate at the latent infection site [23]. This intracellular survival, even a temporary one, allowed the selection of the most resistant bacteria and could have consequences on bacterial behavior and especially on their virulence.

Another important step for bacterial virulence is biofilm formation. Biofilms can be initiated in two different situations: directly when the bacteria get in contact with biotic or abiotic support or indirectly once the bacteria are released from their hosting cell after their first internalization. The main goal of the current study is the comparison of biofilm formation before and after internalization. In our experiments, all strains formed biofilm even if the commensal strains initially formed less biofilm biomass than the BPI strains, confirming previous results [12]. Interestingly, the commensal strains developed more biofilm after internalization with a very high value. In detail, the intracellular bacteria of three skin strains of six formed a significant higher biofilm and one more strain tended to possess the same capacity. On the contrary, the BPI strains formed equal amounts of biofilm pre- and post-internalization, except for strain BPI 4.

The more robust biofilms formed by the four commensal strains seemed to be a non-common phenomenon as two of the four strains kept the phenotype after culture and subcultures, but with lower values, which underlines the possibility of an adaptive and non-hereditary response. As observed, the internalization had no impact on the antibiotic sensibility of the strains, which supported the hypothesis of a temporary change.

Using fluorescent staining, an increase in the live bacteria proportion within the biofilm after internalization was observed regardless of the isolates’ origins. These results suggested that the bacterial stress felt during internalization could lead to a stress adaptation and a better survival of bacteria within the biofilm. This could enhance the biofilm mechanisms in the commensal strains already set up in the BPI strains [10]. *C. acnes* within biofilms presents upregulated, stress-induced genes and CAMP virulent factors involved in host colonization [11,25]. This finding could explain why the biofilm capacity is increased in commensal strains post-internalization. We supposed that the matrix englobing the bacteria could have a different composition after internalization due to the ignition of a “biofilm” program [10]. It would be interesting to quantify the extracellular DNA, proteins and polysaccharides, which compose the biofilm matrix pre- and post-internalization [26].

Finally, in the clinical context, *C. acnes* adhere to prostheses, mostly made of titanium. Looking at the adhesion of the commensal bacteria on titanium, the biofilm formation was greatly increased on the titanium support as compared to the plastic one. Once again, more biofilm was formed with bacteria that are intracellular. This showed that *C. acnes* is strongly attracted to titanium, underlining the clinical results.

No link was identified between the MLST profiles and internalization rates or biofilm behavior. However, two BPI strains (BPI8 and BPI9) belonged to the phylotype IB CC5, whereas this phylotype was not identified in our commensal strains panel. This finding corroborated previous data published by Aubin et al. on the high frequency of this phylotype in infective strains (corresponding to IB CC36 phylotype in Aubin et al., with the MLST9 detection profile [27].

Further investigations will be necessary to decipher the mechanism of interaction between the bone cells and bacteria. It might be supported by a conservative motif, such as the fibronectin-binding protein necessary for internalization of *S. aureus* [8,28], with a different level of expression according to the strain and its microenvironment without a genetic background link [29].

It will be interesting to understand the modifications undergone by the bacteria during internalization (affect metabolism, stress proteins, etc.). Indeed, genetic modification or a punctual mutation could modify the expression of adhesins and so the strain pathogenicity, leading to the transformation of commensal *C. acnes* into pathogens [30]. Therefore, interleukins and chemokines production, reflecting inflammatory processes, will have to be measured to comprehend the bone cells response [21]. These findings are essential to enhance the prevention and the management of the orthopedic implant-associated infections. It is of great importance to gain comprehensive knowledge of the infection pathogenesis thanks to this new approach of research study.

## 5. Conclusions

The commensal *C. acnes* strains’ internalization by osteoblast-like cells had an impact on their biofilm formation capacity, unlike the BPI strains. This effect was amplified on a titanium support. Moreover, all the strains, after internalization, showed an increase in the live proportion within biofilms.

## Figures and Tables

**Table 1:** Phylotype and multilocus sequence typing (MLST) profiles of the *C. acnes* commensal and Bone and Prosthesis Infection (BPI) pathogenic strains.

Isolate Name	Clinical Source	Sequence Typing	Clonal Complex MLST	Phylotype
**C2**	**Skin**	107	CC107	IC
**C5**	**Skin**	1	CC1	IA1
**C7**	**Skin**	49	CC1	IA1
**C8**	**Skin**	95	singleton	IA1
**C9**	**Skin**	46	singleton	IA1
**C18**	**Skin**	143	CC1	IA1
**BPI1**	**Shoulder prosthesis**	20	CC1	IA1
**BPI2**	**Shoulder prosthesis**	1	CC1	IA1
**BPI3**	**Shoulder prosthesis**	20	CC1	IA1
**BPI4**	**Spine material**	21	CC4	IA1
**BPI5**	**Spine material**	1	CC1	IA1
**BPI6**	**Shoulder prosthesis**	51	singleton	IB
**BPI7**	**Hip prosthesis**	55	CC4	IA1
**BPI8**	**Shoulder prosthesis**	152	CC5	IB
**BPI9**	**Tibia material**	152	CC5	IB

## Supplementary Materials

The following are available online at https://www.mdpi.com/2076-2607/8/9/1409/s1, Figure S1: Planktonic growth of *C. acnes* commensal strains, Figure S2: Cytotoxicity after 3 H of *C. acnes*/osteoblast-like cells interaction.
