# Supplementary material for: Cutibacterium acnes Biofilm Study during Bone Cells Interaction
Source: Microorganisms. 2020 Sep 12;8(9):1409. doi: 10.3390/microorganisms8091409 (PMC7564252; doi:10.3390/microorganisms8091409)
Supplement: Supplementary file 1 [file microorganisms-08-01409-s001.pdf]

Article

Cutibacterium acnes biofilm modification during bone cells interaction

Céline Mongaret [1,2,*], Jennifer Varin-Simon [1], Fabien Lamret [1], Taghrid S. El-Mahdy [1,3,4], Lucien Brasme [5], Véronique Vernet-Garnier [5], Sophie C. Gangloff [1], Xavier Ohl [1,6] and Fany Reffuveille [1]

1 EA 4691 Biomatériaux et inflammation en site osseux (BIOS), Université de Reims Champagne-Ardenne, SFR Cap Santé (FED 4231), 51100 Reims, France; celine.mongaret@univ-reims.fr (C.M.); jennifer.varin-simon@univ-reims.fr (J.V.-S.); fabien.lamret@univ-reims.fr (F.L.); sata186@hotmail.com (T.S.E.-M.); sophie.gangloff@univ-reims.fr (S.C.G.); xohl@chu-reims.fr (X.O.); fany.reffuveille@univ-reims.fr (F.R.)
2 Service Pharmacie, Centre Hospitalier Universitaire de Reims (CHU Reims), 51100 Reims, France
3 Department of Microbiology and Immunology, Faculty of Pharmacy, Helwan University, 11795 Cairo, Egypt
4 Department of Microbiology, Faculty of Pharmacy, Ahram Canadian University, 12585 Cairo, Egypt
5 Laboratoire de Bactériologie-Hygiène, Centre Hospitalier Universitaire de Reims (CHU Reims), 51100 Reims, France; lbrasme@chu-reims.fr (L.B.); vvernetgarnier@chu-reims.fr (V.V.-G.)
6 Service d'Orthopédie et Traumatologie, Centre Hospitalier Universitaire de Reims (CHU Reims), 51100 Reims, France
* Correspondence: celine.mongaret@univ-reims.fr

Received: 30 July 2020; Accepted: 10 September 2020; Published: 12 September 2020

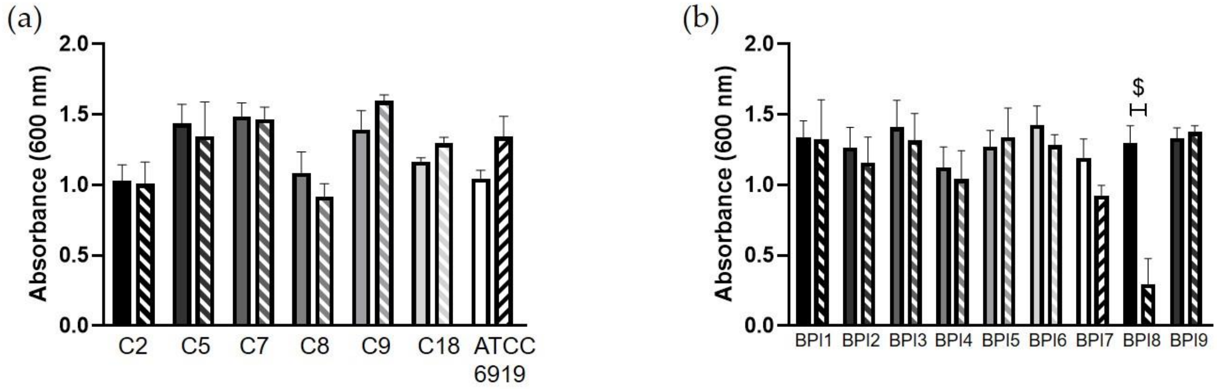

Figure S1: Planktonic growth of *C. acnes* commensal strains (a), before (full bars) and after (hatched bars) internalization and of *C. acnes* isolated from BPIs (b) before (full bars) and after (hatched bars) internalization. *C. acnes* strains were cultivated in Brain Heart Infusion (BHI) broth (BioRad) under anaerobic condition using the GenBox system (Biomerieux) at 37°C during five days. ($, p<0.05).

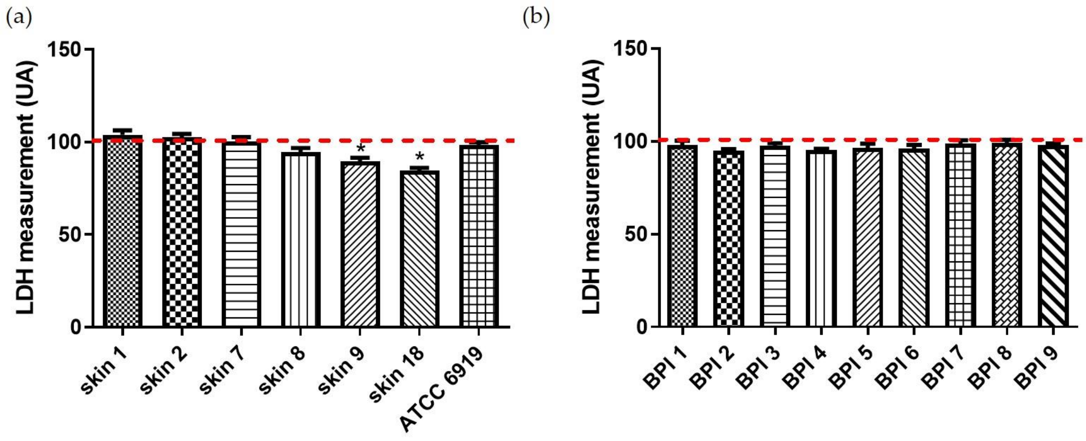

Figure S2: Cytotoxicity of osteoblast-like cells after 3 h of interaction between commensal *C. acnes* or *C. acnes* isolated form BPIs and SaOS2 cells. Lactate deshydrogenase (LDH) release in cells supernatants. LDH release measurement normalized on cells without bacteria, 100% corresponding to the measurement of cells without bacteria.
